# Weight loss efficiency and safety of tirzepatide: A Systematic review

**DOI:** 10.1371/journal.pone.0285197

**Published:** 2023-05-04

**Authors:** Fei Lin, Bin Yu, Baodong Ling, Guangyao Lv, Huijun Shang, Xia Zhao, Xiaoling Jie, Jing Chen, Yan Li

**Affiliations:** 1 Department of Pharmacy, The First Affiliated Hospital of Chengdu Medical College, Chengdu, China; 2 Clinical Medical College, Chengdu Medical College, Chengdu, China; 3 Department of Pharmacy, Mianyang Central Hospital, Mianyang, China; 4 School of Pharmacy, Chengdu Medical College, Chengdu, China; 5 Department of Pharmacy, Binzhou Medical University Hospital, Binzhou, China; 6 School of Pharmacy, Collaborative Innovation Center of Advanced Drug Delivery System and Biotech Drugs in Universities of Shandong, Key Laboratory of Molecular Pharmacology and Drug Evaluation, Ministry of Education, Yantai University, Yantai, China; Universitair Kinderziekenhuis Koningin Fabiola: Hopital Universitaire des Enfants Reine Fabiola, BELGIUM

## Abstract

**Objective:**

Tirzeptide is a novel glucagon-like peptide-1 receptor (GLP-1) and glucose-dependent insulinotropic polypeptide (GIP) drug, which shows good efficiency for weight loss. Therefore, we aim to investigate the efficacy and safety of tirzepatide for weight loss in type 2 diabetes mellitus (T2DM) and obesity patients in this meta-analysis study.

**Methods:**

Cochrane Library, PubMed, Embase, Clinical Trials, and Web of Science were searched from inception to October 5, 2022. All randomized controlled trials (RCTs) were included. The odds ratio (OR) was calculated using fixed-effects or random-effects models by Review Manager 5.3 software.

**Results:**

In total, ten studies (12 reports) involving 9,873 patients were identified. A significant loss body weight in the tirzepatide group versus the placebo by -9.81 kg (95% CI (-12.09, -7.52), GLP-1 RAs by -1.05 kg (95% CI (-1.48, -0.63), and insulin by -1.93 kg (95% CI (-2.81, -1.05), respectively. In sub-analysis, the body weight of patients was significantly reduced in three tirzepatide doses (5 mg, 10 mg, and 15 mg) when compared with those of the placebo/GLP-1 RA/insulin. In terms of safety, the incidence of any adverse events and adverse events leading to study drug discontinuation was higher in the tirzepatide group, but the incidence of serious adverse events and hypoglycaemia was lower. Additionally, the gastrointestinal adverse events (including diarrhea, nausea, vomiting and decreased appetite) of tirzepatide were higher than those of placebo/basal insulin, but similar to GLP-1 RAs.

**Conclusion:**

In conclusion, tirzeptide can significantly reduce the weight of T2DM and patient with obesity, and it is a potential therapeutic regimen for weight-loss, but we need to be vigilant about its gastrointestinal reaction.

## Introduction

Obesity is a metabolic disease, which is related to a variety of chronic diseases in addition to affecting the quality of life [1]. Recent statistics indicate that overweight/obesity and its relentless global rise, with the number of people with excess body weight reaching > 2 billion, approximately 30% of the world population [2]. Some researchers reckon that overweight and obesity are major risk factors for cardiovascular disease [3]. Thus, weight loss can reduce the incidence of cardiovascular events and all-cause mortality in cardiovascular patients [3, 4], and lessen the incidence of diabetes [5, 6]. Currently, a growing number of drugs are used for weight loss, such as glucagon-like peptide-1 receptor agonists (GLP-1 RAs). Liraglutide was the first GLP-1RAs to be approved by the U.S. Food and Drug Administration (FDA) and European Medicines Agency (EMA) for the treatment of obesity [7]. Additionally, more evidence supports the use of the GLP-1RAs semaglutide in people with obesity without type 2 diabetes mellitus (T2DM) [8].

As time goes on, an increasing number of drugs have been developed for the treatment of T2DM or obesity. In recent years, Glucagon-like peptide-1 receptor (GLP-1) and glucose-dependent insulinotropic polypeptide (GIP) are known as incretins among the many hormones in the body that has attracted the attention of researchers, which can promote insulin release after meals, lowering blood sugar and making the body more sensitive to insulin [9, 10]. Moreover, it also contributed to weight loss by slowing gastric emptying. GLP-1RAs are now considered the choice of injectable therapy for many people with T2DM and obesity, with several members of the class having weight loss efficacy [11–13]. Building on that concept, the combined GIP and GLP-1 RAs have been proposed as a novel therapeutic option for T2DM and obesity.

Tirzepatide (LY3298176, Mounjaro) is the first dual GIP and GLP-1 RAs for the treatment of T2DM, obesity, and nonalcoholic steatohepatitis [14]. It is a first-in-class GLP-1/GIP receptor agonists that FDA approved on May 13, 2022, to improve blood sugar control in adults with T2DM as an adjunct to diet and exercise [15]. Tirzepatide can lower the hemoglobin A1C level more than other medications to which it was compared [16, 17]. At the same time, there is growing evidence that tirzepatide plays a role in the weight loss of T2DM patients. Furthermore, another study showed that tirzepatide did not increase the risk of major cardiovascular events in participants with T2DM versus controls [18]. Tirzepatide also supported substantial weight loss in a recent clinical trial, potentially supporting its use as an obesity treatment [19].

In this paper, we performed a comprehensive systematic review and meta-analysis of all currently available randomized controlled trials (RCTs) of tirzepatide in individuals with T2DM and obesity to evaluate weight loss and adverse events when they were treated with tirzepatide.

## Methods

### Study search and selection

To conduct our study, we systematically searched PubMed, EMBASE, Cochrane library, Web of Science, and Clinical Trails databases from their inception to October 5, 2022, in the English language. "Tirzepatide" [MeSH] OR "LY3298176" OR "Mounjaro" were among the search phrases used. According to the inclusion and exclusion criteria, two researchers independently read the title and abstract of the literature for preliminary screening and also read the full text of literature that potentially met the inclusion criteria. Any disagreement was discussed and decided by the third researcher.

Studies were included for this meta-analysis if they met the following criteria: only RCT; adults of obesity patients with or without T2DM; tirzepatide is the intervention drug; comparison is placebo or antidiabetic; and outcome of efficacy and safety. Authorship; year of publication; randomization; intervention; and patient number; study design; study duration; study site; study population; therapy duration; body weight; and risk of AEs were extracted from all included studies.

### Outcome indicators and the risk of bias assessment

The primary outcome indicators included body weight, glycosylated hemoglobin, type A1C (HbA1c) and the incidence of any AEs. The secondary outcome indicators included the incidence of SAEs, AE leading to study drug discontinuation, hypoglycemia, and other AEs. The Cochrane Collaboration bias assessment tool was used to evaluate the risk bias of the included studies by two researchers independently [20]. According to the tool the risk was categorized as “high risk”, “low risk”, or “unclear”. Review Manager 5.3 was used to carry out quality assessment and an investigation of publication bias.

### Statistical analysis

Review Manager 5.3 was utilized to perform statistical analysis. The mean difference (MD) was used as the effect analysis statistic for continuous measurement data; Oddi ratio (OR) was used as the effect analysis statistic for dichotomous variables, and 95%CI was considered for each effect. Statistical heterogeneity between the results was analyzed by Chi-square (χ^2^) test, and the heterogeneity was quantitatively judged by I^2^. When I^2^ ≤ 50% and *P* > 0.1, the fixed effect model was applied, and when I^2^ > 50% and *P* < 0.1, the random effect model was applied. Additionally, we also investigated the source of heterogeneity with a sensitivity analysis when I^2^ was higher than 50%. The meta-analysis level was set as 0.05.

## Results

### Searching results and study characteristics

**Fig 1:**
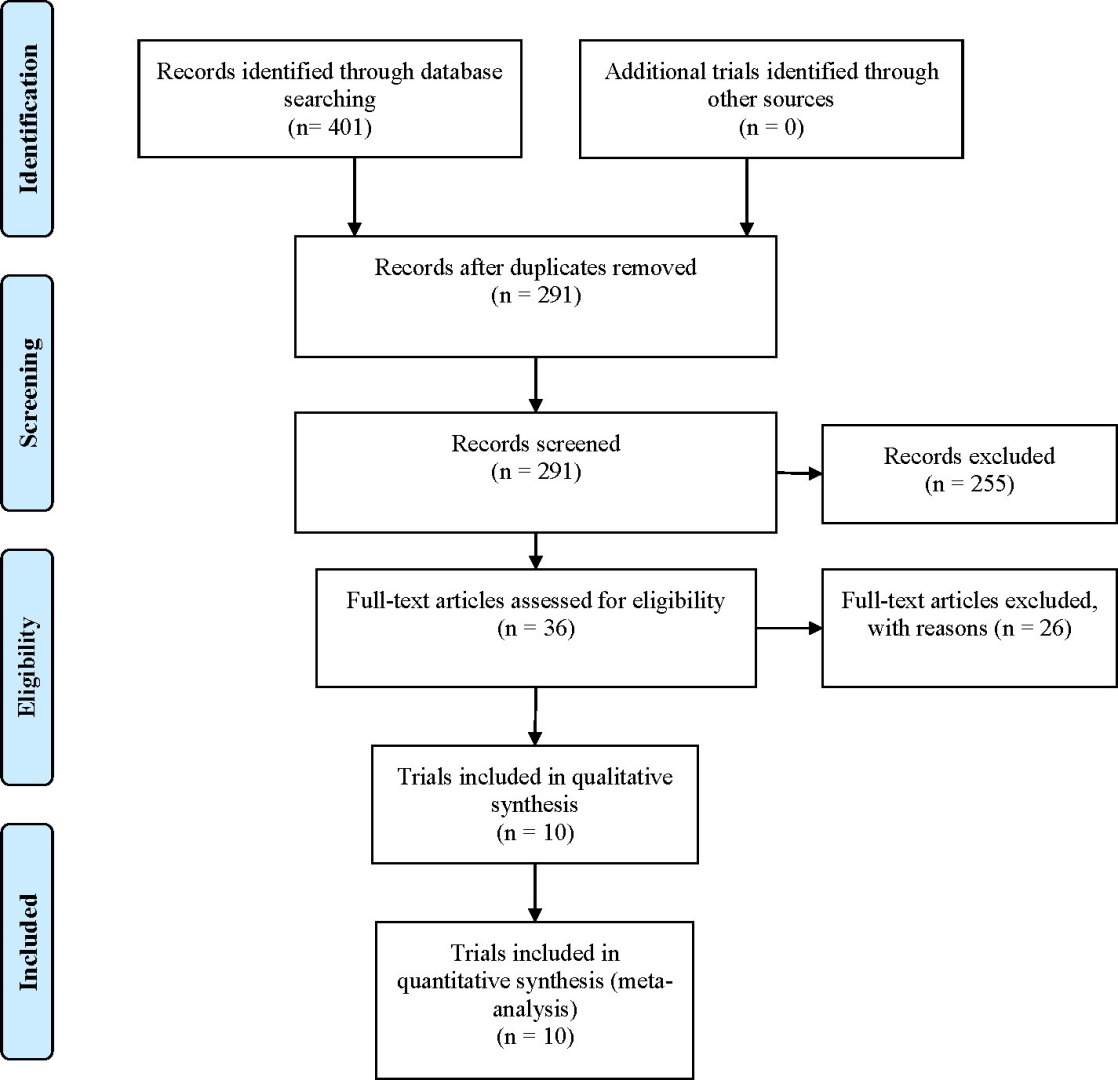
Flow diagram of studies searched in this meta-analysis.

The initial 401 articles were searched, including Cochrane library (n = 38), PubMed (n = 74), Embase (n = 156), Clinical Trails (n = 25) and Web of Science (n = 108). The duplicate literature (n = 140) was first removed with EndNote X8 software, then the rest literature was further read for screening, and finally, the 10 studies that conformed to the inclusion criteria were included. A total of 9873 T2DM patients were involved. All studies were published in English. In this study, three tirzepatide doses has been giving (5 mg, 10 mg and 15 mg, subcutaneous injection, once a week), and a comparator, including placebo (two study by Frías [21, 22], SURPASS-1 [23], SURPASS-5 [24], SURMOUNT-1 [19], study by Heise [25], basal insulin [10 U/day insulin degludec (SURPASS–3) [26], and 10 U/day insulin glargine (SURPASS-4) [27]], GLP-1 RAs [1mg semaglutide (SURPASS-2) [28], and 1.5mg dulaglutide [21], 0.75 mg dulaglutide (SURPASS J–mono) [29]]. The study by Frias [21] with five groups, including tirzepatide (5 mg, 10 mg, 15 mg), placebo, and dulaglutide (1.5 mg) groups. Another one study by Frías [22] had four groups, we just including tirzepatide (15^−2^ mg) and placebo. The 15^−2^ mg tirzepatide dose-escalation regimens were 2.5 mg weeks 0–3; 7.5 mg weeks 4–7; and 15 mg weeks 8–11. Meanwhile, the duration of intervention in 4 studies was 40 weeks, 4 studies was 52 weeks, one study was 26 weeks, and another duration was 12 weeks. Ten studies were published from 2018 to 2022. The literature screening process and results are shown in Fig 1. Table 1 shows the baseline characteristics of the selected studies.

**Table 1:** General baseline of included studies.

Study, year published	Intervention	Patient number	Study duration	Therapy duration	Study population	Study design	Study site	Male (%)	Mean ±S.D. age
Frías JP, et al. 2018	5mg	55	Between May, 2017 and March, 2018	26–week	18–75 years with 2 type diabetes for at least 6 months, HbA1c (7.0–10.5), BMI 23–50 kg/m².	phase 2b	47 sites in 4 countries	34 (62)	57.9 ± 8.2
	10mg	51						30 (59)	56.5 ± 9.9
	15mg	53						22 (42)	56.0 ± 7.6
	1.5mg dulaglutide	54						24 (44)	58.7 ± 7.8
	placebo	51						29 (57)	56.6 ± 8.9
Frías JP, et al. 2020	15mg	28	Between November, 2017 and April, 2018	12–week	Type 2 diabetes for at least 6 months HbA1c 7.0–10.5, BMI 23–45 kg/m2.	phase 2	13 sites in United States	23 (82.1)	56.6 ± 9.21
	placebo	26						12 (46.2)	56.0 ± 10.13
Rosenstock J, et al. 2021 (SURPASS–1)	5mg	121	Between June, 2019 and Oct, 2020	40–week	≥18 years with type 2 diabetes. HbA1c 7.0–9.5, BMI≥23 kg/m², and stable weight (±5) during the previous 3 months	phase 3	52 sites in 4 countries	56 (46)	54.1 ± 11.9
	10mg	121						72 (60)	55.8 ± 10.4
	15mg	121						63 (52)	52.9 ± 12.3
	placebo	115						56 (49)	53.6 ± 12.8
Frías JP, et al. 2021 (SURPASS–2)	5mg	470	Between July, 2019 and February, 2021	40–week	≥18 years with type 2 diabetes, metformin≥ 1500 mg/d. HbA1c 7.0–10.5, BMI ≥25 kg/m², stable weight (±5) during the previous 3 months.	open–label, phase 3	128 sites in 8 countries	205 (43.6)	56.3 ±10.0
	10mg	469						238(50.7)	57.2 ±10.5
	15mg	470						214(45.5)	55.9 ±10.4
	1 mg semaglutide	469						225(48.0)	56.9 ±10.8
Ludvik B, et al. 2021 (SURPASS–3)	5mg	358	Between April, 2019 and Jan, 2021	52–week	≥18 years and type 2 diabetes, HbA1c 7.0–10.5, metformin alone or combination with an SGLT2 inhibitor for at least 3 months, BMI ≥ 25 kg/m², and stable weight (±5) during the previous3 months.	open label, phase 3	122 sites in 13 countries	200 (56)	57.2 ± 10.1
	10mg	360						195 (54)	57.4 ± 9.7
	15mg	359						194 (54)	57.5 ± 10.2
	degludec	360						213 (59)	57.5 ± 10.1
Prato SD, et al. 2021 (SURPASS–4)	5mg	329	Between Nov, 2018 and April, 2021	52–week	≥18 years with type 2 diabetes, HbA1c 7.5–10.5, three oral glucose–lowering medications either alone or in any combination, BMI ≥ 25 kg/m², and stable weight (≤5) during the previous 3 months.	open–label, phase 3	187 sites in 14 countries	198 (60)	62.9 ± 8.6
	10mg	328						209 (64)	63.7 ± 8.7
	15mg	338						203 (60)	63.7 ± 8.6
	glargine	1000						636 (64)	63.8 ± 8.5
Dahl D, et al. 2022 (SURPASS–5)	5mg	116	Between August, 2019 and January, 2021	40–week	adults with type 2 diabetes, HbA1c 7.0–10.5, BMI ≥ 23 kg/m², insulin glargine (>20 IU/d or >0.25 IU/kg/d) with or without metformin (≥1500 mg/d).	phase 3	45 sites in in 8 countries	60 (10)	62 ± 10
	10mg	119						72 (61)	60 ± 10
	15mg	120						65 (54)	61 ± 10
	placebo	120						66 (55)	60 ± 10
Inagaki N, et al. 2022 (SURPASS J–mono)	5mg	159	Between May, 2019 and March, 2021	52–week	≥20 Years with type 2 diabetes, HbA1c 7.0–10.0, BMI ≥23 kg/m², stable weight (±5) during 3 months preceding	phase 3	Japan	113 (71.1)	56.8 ± 10.1
	10mg	158						119 (75.3)	56.2 ± 10.3
	15mg	160						132 (82.5)	56.0 ± 10.7
	dulaglutide	159						117 (73.6)	57.5 ± 10.2
Heise T, et al. 2022	15 mg	45	Between June 28, 2019, and April 8, 2021,	28-week	20–74 years, type 2 diabetes for at least 6 months, and were being treated with lifestyle advice and stable doses of metformin, with or without one additional stable dose of another oral anti-hyperglycaemic medicine	phase 1	2 sites in Germany	31 (69.0)	61.1 ± 7.1
	Semaglutide 1 mg	44						34 (77.0)	63.7 ± 5.9
	Placebo	28						21 (75.0)	60.4 ± 7.6
Jastreboff, AM, et al. 2022 (SURMOUNT-1)	5mg	630	Between December 2019 and April 2022	72-week	≥18 years, BMI ≥ 30 kg/m² or BMI ≥ 27 kg/m²or more and at least one weight-related complication.	phase 3	119 sites in 9 countries	204 (32.3)	45.6±12.7
	10mg	636						209 (32.9)	44.7±12.4
	15mg	630						205 (32.5)	44.9±12.3
	placebo	643						207 (32.2)	44.4±12.5

### Quality assessment

**Fig 2:**
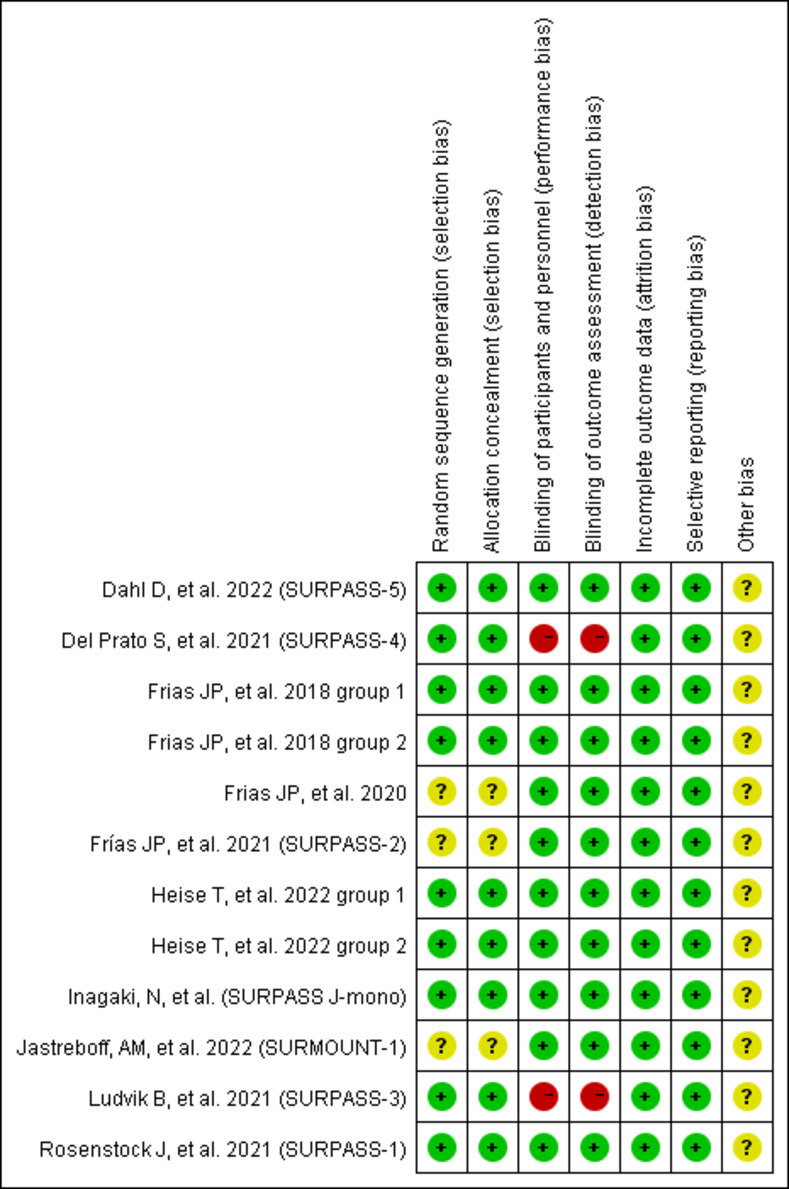
Graphs of risk of bias for studies.

**Fig 3:**
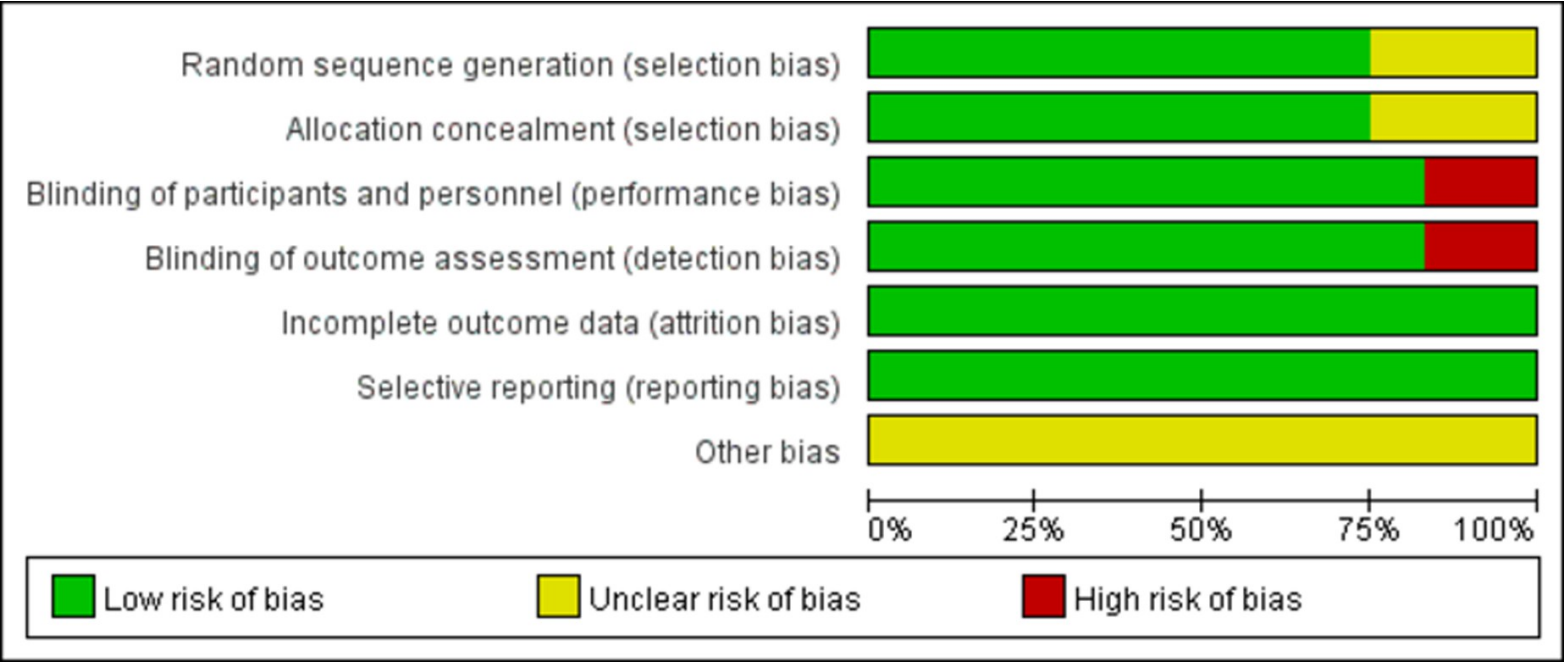
Quality assessment for risk of bias for studies.

The results of the quality assessment of 10 studies are furnished in Fig 2. Five RCTs described the detailed randomization methods, allocation concealment, blinding of participants and personnel, incomplete outcome data, and other biases. Three RCTs did not have detail randomization methods and allocation concealment. Two RCTs are open-label and have a high bias risk for research. The risks of study design bias was shown in Fig 3.

### Efficacy analysis

**Fig 4:**
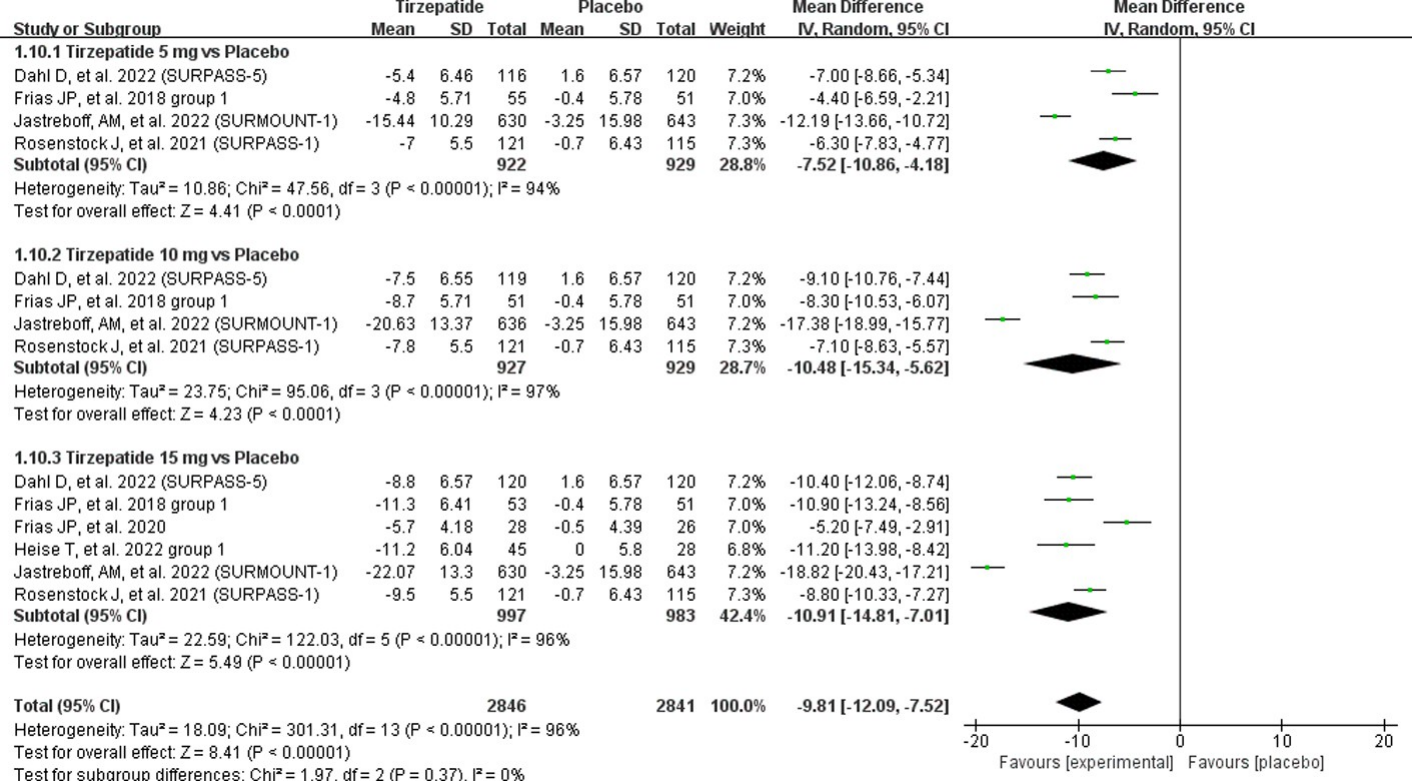
Effect of tirzepatide vs placebo on body weight.

In this meta-analysis, the included 10 RCT studies displayed varying degrees of weight loss efficacy. Over all, meta-analysis showed a significant reduction in body weight in the tirzepatide group versus the placebo group by -9.81 kg (95% CI (-12.09, -7.52). There were three doses investigated compared to the placebo group were affected significantly reduced the body weight of patients [5 mg: MD = -7.52 kg, 95% CI (-10.86, -4.18), *P* < 0.0001; I^2^ = 94%; 10 mg: MD = -10.48 kg, 95% CI (-15.34, -5.62), *P* < 0.0001; I^2^ = 97%; 15 mg: MD = -10.91 kg, 95% CI (-14.81, -7.01), *P* < 0.00001; I^2^ = 96%] (Fig 4). The sensitivity analysis excluding the SURMOUNT-1 [19] trial showed that statistical heterogeneity decreased from 94% to 43%, 97% to 35%, and 96% to 78%, respectively.

**Fig 5:**
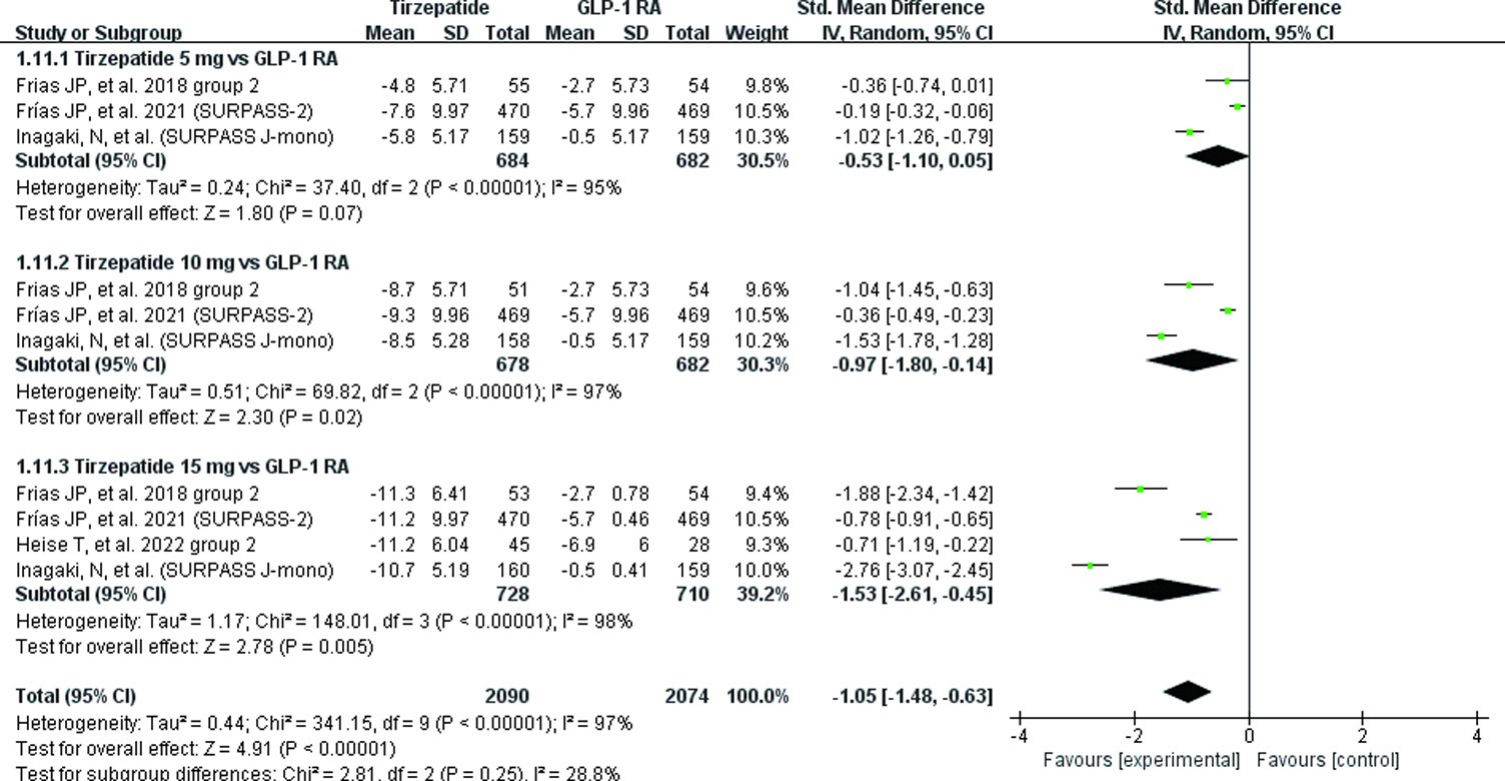
Effect of tirzepatide vs GLP-1 RAs (semaglutide and dulaglutide) on body weight.

The body weight of patients was significantly reduced 1.05 kg (95% CI (-1.48, -0.63) when compared with GLP-1 RAs group. There were three doses investigated [5 mg: MD = -0.53, 95% CI (-1.10, -0.05), *P* = 0.07; I^2^ = 95%; 10 mg: MD = -0.97, 95% CI (-1.80, -0.1), *P* = 0.02; I^2^ = 97%; 15 mg: MD = -1.53, 95% CI (-2.61, -0.45), *P* = 0.005; I^2^ = 98%] (Fig 5). The sensitivity analysis removing SURPASS J-mono [30] trial showed that statistical heterogeneity decreased from 95% to 0%, 97% to 90%, and 98% to 90%, respectively.

**Fig 6:**
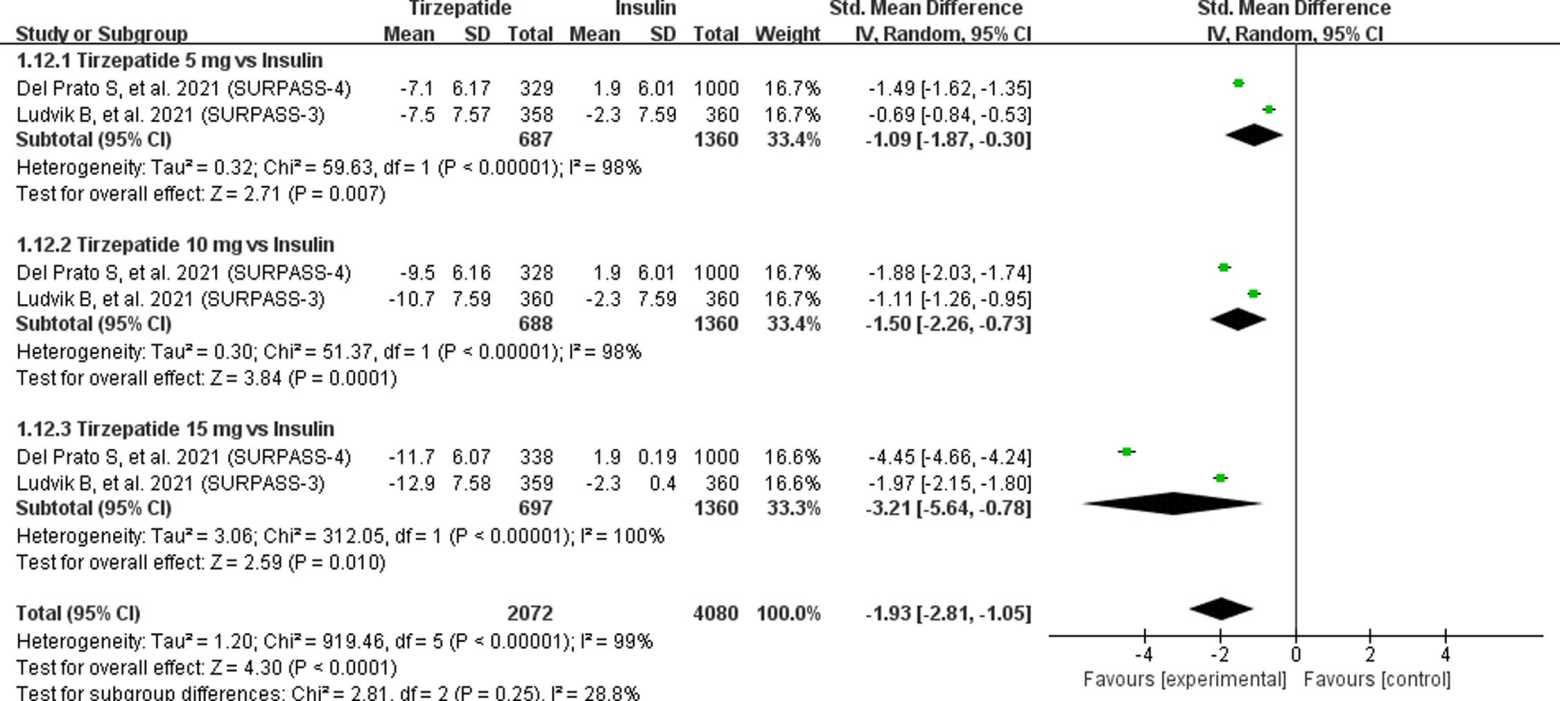
Effect of tirzepatide vs insulin (insulin degludec and insulin glargine) on body weight.

The body weight of patients was significantly decreased 1.93 kg (95% CI (-2.81, -1.05) when compared with insulin group. Three doses were tested [5 mg: MD = -1.09, 95% CI (-1.87, -0.30), *P* = 0.007; I^2^ = 98%; 10 mg: MD = -1.50, 95% CI (-2.26, -0.73), *P* = 0.0001; I^2^ = 98%; 15 mg: MD = -3.21, 95% CI (-5.64, -0.78), *P* = 0.01; I^2^ = 100%] significantly decreased the body weight of patients when compared with insulin group (Fig 6). Initially, the heterogeneities of three tirzepatide doses were observed to be high, but when we removed any one study, the heterogeneities in both groups did not decrease remarkably. Consistently, compared with placebo, GLP-1 RAs and insulin, more participants receiving any of the three tirzepatide doses had reductions in body weight of at least 5%, 10%, or 15% (Table 2).

**Table 2:** Meta-analysis results for tirzepatide vs placebo, GLP-1 RAs (semaglutide and dulaglutide) and basal insulin (insulin degludec and insulin glargine) for weigh loss.

Intervention	Comparator	No. of participants with outcome/participants analysed		OR (95% CI)	I^2^ (%)	*P* value
		Tirzepatide arm	Comparator arm			
≥5% weight loss						
Tirzepatide 5 mg	Placebo	699/922	245/929	11.93 [9.39, 15.15]	0	< 0.00001
	GLP-1 RAs	428/684	282/682	3.97 [0.97, 16.26]	95	0.06
	Basal insulin	438/687	100/1360	22.66 [15.64, 32.84]	42	< 0.00001
Tirzepatide 10 mg	Placebo	761/927	245/929	17.33 [13.43, 22.35]	34	< 0.00001
	GLP-1 RAs	522/678	282/682	9.43 [1.58, 56.18]	97	0.01
	Basal insulin	542/688	100/1360	48.54 [27.20, 86.65]	72	< 0.00001
Tirzepatide 15 mg	Placebo	780/924	245/929	21.41 [16.37, 28.00]	38	< 0.00001
	GLP-1 RAs	552/683	282/682	11.02 [1.66, 73.26]	97	0.01
	Basal insulin	595/697	100/1360	75.38 [50.05, 113.54]	40	< 0.00001
≥10% weight loss						< 0.00001
Tirzepatide 5 mg	Placebo	502/922	214/929	15.82 [3.29, 76.10]	73	0.0006
	GLP-1 RAs	169/525	118/523	1.64 [1.24, 2.16]	0	0.0004
	Basal insulin	249/687	25/1360	27.27 [17.59, 42.25]	42	< 0.00001
Tirzepatide 10 mg	Placebo	613/927	214/929	33.97 [5.62, 205.33]	79	0.0001
	GLP-1 RAs	240/520	118/523	2.95 [2.25, 3.86]	52	< 0.00001
	Basal insulin	365/688	25/1360	54.19 [35.06, 83.75]	34	< 0.00001
Tirzepatide 15 mg	Placebo	649/924	214/929	37.79 [7.68, 186.01]	74	< 0.00001
	GLP-1 RAs	288/523	118/523	4.28 [3.27, 5.61]	0	< 0.00001
	Basal insulin	464/697	25/1360	96.07 [62.15, 148.50]	15	< 0.00001
≥15% weight loss						
Tirzepatide 5 mg	Placebo	329/922	57/929	9.95 [7.30, 13.55]	0	< 0.00001
	GLP-1 RAs	73/525	38/523	2.07 [1.37, 3.14]	0	0.0006
	Basal insulin	89/687	5/1360	43.49 [16.98, 111.43]	0	< 0.00001
Tirzepatide 10 mg	Placebo	483/927	57/929	22.31 [16.25, 30.64]	0	< 0.00001
	GLP-1 RAs	124/520	38/523	3.99 [2.71, 5.88]	38	< 0.00001
	Basal insulin	176/688	5/1360	95.35 [37.61, 241.73]	32	< 0.00001
Tirzepatide 15 mg	Placebo	516/924	57/929	27.14 [19.66, 37.47]	0	< 0.00001
	GLP-1 RAs	182/523	38/523	6.88 [4.72, 10.04]	0	< 0.00001
	Basal insulin	272/697	5/1360	174.26 [69.74, 435.43]	33	< 0.00001

The changes of HbA1c of patients was also collected. When compared with placebo, tirzepatide can significantly reduce the HbA1c of patients [5 mg: MD = -1.55%, 95% CI (-1.72, -1.39), P < 0.00001; I^2^ = 85%; 10 mg: MD = -1.75%, 95% CI (-1.92, -1.58), P < 0.00001; I^2^ = 71%; 15 mg: MD = -1.87%, 95% CI (-2.03, -1.70), P < 0.00001; I^2^ = 86%]. The same results were found in comparing tirzepatide with GLP-1 RAs group and insulin group, the level of HbA1c of all patients was significantly reduced [GLP-1 RAs: 5 mg: MD = -0.51%, 95% CI (-0.62–0.39), P < 0.00001; I^2^ = 96%; 10 mg: MD = -0.73%, 95% CI (-0.85, -0.62), P < 0.00001; I^2^ = 96%; 15 mg: MD = -0.89%, 95% CI (-1.00, -0.77), P < 0.00001; I^2^ = 97%; Insulin: 5 mg: MD = -0.71%, 95% CI (-0.80, -0.63), P < 0.00001; I^2^ = 81%; 10 mg: MD = -0.94%, 95% CI (-1.03, -0.85), P < 0.00001; I^2^ = 50%; 15 mg: MD = -1.10%, 95% CI (-1.18, -1.01), P < 0.00001; I^2^ = 30%].

### Safety analysis

For the safety, meta-analysis showed a significant difference the incidence of any adverse events between tirzepatide group and placebo group [OR = 1.59, 95% CI (1.29, 1.95), P < 0.00001, I² = 53%], GLP-1 RAs group [OR = 1.15, 95% CI (1.00, 1.32), P = 0.05, I² = 0%], and basal insulin [OR = 1.55, 95% CI (1.25, 1.91), P < 0.0001, I² = 69%], respectively. In the sub-analysis, the incidence of any adverse events was lower in the tirzepatide group than in the placebo group and basal insulin. But when compared to GLP-1 RAs, there was no significant difference in the tirzepatide 5 mg [OR = 1.01, 95% CI (0.80, 1.28), *P* = 0.92] and 10mg [OR = 1.17, 95% CI (0.92, 1.48), *P* = 0.2] groups (Table 3). Additionally, there was a statistically significant difference in the incidence of adverse events leading to study drug discontinuation between tirzepatide and placebo, between tirzepatide (15mg) and GLP-1 RAs, and between tirzepatide (5mg) and basal insulin. However, no significant statistics were found between tirzepatide and GLP-1 RAs (5mg and 10 mg) or between tirzepatide (10mg and 15 mg) and basal insulin.

**Table 3:** The results of safety in meta-analysis.

Intervention	Comparator	Studies (n)	Tirzepatide arm (n)	Comparator arm (n)	I^2^ (%)	Effect Estimate	*P* value
Any Adverse events							
Tirzepatide 5 mg	Placebo	4	718/922	647/929	0	1.55 [1.25, 1.91]	< 0.0001
	GLP-1 RAs	3	478/684	475/682	0	1.01 [0.80, 1.28]	0.92
	Basal insulin	2	451/687	872/1360	0	1.23 [1.01, 1.50]	0.04
Tirzepatide 10 mg	Placebo	4	722/927	647/929	62	1.48 [0.98, 2.23]	0.06
	GLP-1 RAs	3	493/678	475/682	0	1.17 [0.92, 1.48]	0.2
	Basal insulin	2	489/688	872/1360	69	1.58 [1.09, 2.29]	0.02
Tirzepatide 15 mg	Placebo	6	780/997	682/983	71	2.06 [1.24, 3.43]	0.005
	GLP-1 RAs	4	553/728	518/726	0	1.28 [1.01, 1.63]	0.04
	Basal insulin	2	522/697	872/1360	74	1.91 [1.26, 2.89]	0.002
Serious adverse events							
Tirzepatide 5 mg	Placebo	4	55/922	59/929	0	0.94 [0.64, 1.38]	0.76
	GLP-1 RAs	3	59/684	30/682	37	2.08 [1.32, 3.28]	0.002
	Basal insulin	2	77/687	215/1360	71	0.94 [0.51, 1.75]	0.85
Tirzepatide 10 mg	Placebo	4	62/927	59/929	0	1.06 [0.73, 1.54]	0.74
	GLP-1 RAs	3	53/678	30/682	0	1.87 [1.17, 2.98]	0.008
	Basal insulin	2	74/688	215/1360	0	0.84 [0.63, 1.13]	0.24
Tirzepatide 15 mg	Placebo	5	45/969	61/957	0	0.72 [0.49, 1.07]	0.11
	GLP-1 RAs	4	55/728	30/726	0	1.91 [1.20, 3.01]	0.006
	Basal insulin	2	67/697	215/1360	77	0.80 [0.39, 1.64]	0.54
Adverse event leading to study drug discontinuation							
Tirzepatide 5 mg	Placebo	4	43/922	25/929	0	1.77 [1.07, 2.92]	0.03
	GLP-1 RAs	2	33/525	25/523	0	1.34 [0.78, 2.29]	0.29
	Basal insulin	2	62/687	59/1360	63	3.09 [1.33, 7.18]	0.009
Tirzepatide 10 mg	Placebo	4	64/927	25/929	0	2.68 [1.68, 4.30]	< 0.0001
	GLP-1 RAs	2	43/520	25/523	72	1.23 [0.30, 5.09]	0.78
	Basal insulin	2	65/688	59/1360	89	3.46 [0.69, 17.43]	0.13
Tirzepatide 15 mg	Placebo	6	74/997	29/983	48	2.57 [1.67, 3.96]	< 0.0001
	GLP-1 RAs	3	54/568	24/567	0	2.40 [1.46, 3.95]	0.0005
	Basal insulin	2	75/697	59/1360	87	4.00 [0.96, 16.79]	0.06
Hypoglycemia (blood glucose <70 mg/dL)							
Tirzepatide 5 mg	Placebo	3	81/292	76/286	44	1.22 [0.76, 1.96]	0.4
	GLP-1 RAs	2	7/525	4/523	0	1.76 [0.51, 6.13]	0.37
	Basal insulin	2	142/687	811/1360	94	0.17 [0.06, 0.48]	0.0008
Tirzepatide 10 mg	Placebo	3	88/291	76/286	51	1.42 [0.89, 2.27]	0.14
	GLP-1 RAs	2	6/520	4/523	25	1.59 [0.44, 5.73]	0.48
	Basal insulin	2	156/688	811/1360	72	0.22 [0.15, 0.34]	< 0.00001
Tirzepatide 15 mg	Placebo	5	92/367	81/340	24	1.25 [0.81, 1.93]	0.3
	GLP-1 RAs	4	17/728	5/726	0	3.29 [1.25, 8.69]	0.02
	Basal insulin	2	178/697	811/1360	86	0.25 [0.14, 0.46]	< 0.00001
Nausea							
Tirzepatide 5 mg	Placebo	4	195/922	74/929	0	3.15 [2.34, 4.17]	< 0.00001
	GLP-1 RAs	3	112/684	112/682	35	1.00 [0.65, 1.56]	0.99
	Basal insulin	2	80/687	29/1360	0	6.38 [4.02, 10.11]	< 0.00001
Tirzepatide 10 mg	Placebo	4	260/927	74/929	0	4.59 [3.46, 6.07]	< 0.00001
	GLP-1 RAs	3	132/678	112/682	77	1.31 [0.62, 2.75]	0.48
	Basal insulin	2	134/688	29/1360	58	11.02 [5.21, 23.31]	< 0.00001
Tirzepatide 15 mg	Placebo	6	281/997	83/983	53	4.19 [2.37, 7.39]	< 0.00001
	GLP-1 RAs	4	168/728	125/726	54	1.51 [0.94, 2.44]	0.09
	Basal insulin	2	161/697	29/1360	0	14.34 [9.30, 22.10]	< 0.00001
Diarrhea							
Tirzepatide 5 mg	Placebo	4	153/922	70/929	39	2.13 [1.30, 3.48]	0.003
	GLP-1 RAs	3	96/684	74/682	63	1.34 [0.97, 1.85]	0.08
	Basal insulin	2	96/687	58/1360	0	3.63 [2.53, 5.19]	< 0.00001
Tirzepatide 10 mg	Placebo	4	180/927	70/929	59	2.59 [1.41, 4.76]	0.002
	GLP-1 RAs	3	104/678	74/682	0	1.50 [1.09, 2.06]	0.01
	Basal insulin	2	125/688	58/1360	0	5.20 [3.69, 7.32]	< 0.00001
Tirzepatide 15 mg	Placebo	6	214/997	78/983	50	2.65 [1.58, 4.44]	0.0002
	GLP-1 RAs	4	104/728	87/726	0	1.23 [0.90, 1.67]	0.19
	Basal insulin	2	130/697	58/1360	0	5.47 [3.90, 7.68]	< 0.00001
Vomiting							
Tirzepatide 5 mg	Placebo	4	59/922	17/929	50	3.67 [2.13, 6.33]	< 0.00001
	GLP-1 RAs	3	42/684	46/682	79	1.16 [0.25, 5.34]	0.85
	Basal insulin	2	37/687	20/1360	0	3.94 [2.19, 7.09]	< 0.00001
Tirzepatide 10 mg	Placebo	4	77/927	17/929	60	4.86 [2.86, 8.27]	< 0.00001
	GLP-1 RAs	3	52/678	46/682	35	1.25 [0.60, 2.59]	0.55
	Basal insulin	2	61/688	20/1360	0	6.77 [3.91, 11.71]	< 0.00001
Tirzepatide 15 mg	Placebo	6	102/997	19/983	37	5.76 [3.51, 9.45]	< 0.00001
	GLP-1 RAs	4	76/728	51/726	69	1.75 [0.66, 4.61]	0.26
	Basal insulin	2	65/697	20/1360	0	7.13 [4.15, 12.26]	< 0.00001
Decreased appetite							
Tirzepatide 5 mg	Placebo	4	83/922	25/929	0	3.60 [2.28, 5.68]	< 0.00001
	GLP-1 RAs	3	68/684	35/682	54	2.39 [1.15, 4.96]	0.02
	Basal insulin	2	51/687	7/1360	0	15.83 [6.93, 36.18]	< 0.00001
Tirzepatide 10 mg	Placebo	4	109/927	25/929	1	4.85 [3.11, 7.56]	< 0.00001
	GLP-1 RAs	3	68/678	35/682	64	2.58 [1.11, 5.99]	0.03
	Basal insulin	2	73/688	7/1360	0	22.72 [9.99, 51.68]	< 0.00001
Tirzepatide 15 mg	Placebo	6	126/997	32/983	25	4.27 [2.84, 6.43]	< 0.00001
	GLP-1 RAs	4	114/728	66/726	79	2.18 [0.86, 5.50]	0.1
	Basal insulin	2	78/697	7/1360	0	23.59 [10.38, 53.61]	< 0.00001
Injection site reactions							
Tirzepatide 5 mg	Placebo	4	29/922	5/929	0	4.78 [1.83, 12.49]	0.001
	GLP-1 RAs	3	14/684	19/682	80	0.78 [0.09, 6.65]	0.82
	Basal insulin	2	2/687	10/1360	0	0.31 [0.07, 1.40]	0.13
Tirzepatide 10 mg	Placebo	4	47/927	5/929	36	6.18 [1.80, 21.17]	0.004
	GLP-1 RAs	3	22/678	19/682	80	1.29 [0.20, 8.15]	0.79
	Basal insulin	2	8/688	10/1360	0	1.13 [0.44, 2.94]	0.8
Tirzepatide 15 mg	Placebo	5	53/969	17/957	81	2.78 [0.45, 17.40]	0.27
	GLP-1 RAs	4	34/728	31/726	83	0.68 [0.07, 6.36]	0.74
	Basal insulin	2	9/697	10/1360	0	1.19 [0.46, 3.06]	0.72
Nasopharyngitis							
Tirzepatide 5 mg	Placebo	3	28/292	39/289	0	0.69 [0.41, 1.16]	0.16
	GLP-1 RAs	2	32/214	32/213	23	1.00 [0.58, 1.70]	0.99
	Basal insulin	2	21/687	87/1360	0	0.47 [0.28, 0.77]	0.003
Tirzepatide 10 mg	Placebo	3	18/291	39/289	0	0.42 [0.24, 0.76]	0.004
	GLP-1 RAs	2	27/209	32/213	31	0.83 [0.48, 1.45]	0.52
	Basal insulin	2	30/688	87/1360	0	0.69 [0.45, 1.07]	0.1
Tirzepatide 15 mg	Placebo	4	33/339	42/317	0	0.70 [0.43, 1.14]	0.15
	GLP-1 RAs	3	32/258	40/257	0	0.76 [0.46, 1.26]	0.29
	Basal insulin	2	31/697	87/1360	0	0.70 [0.45, 1.07]	0.1

GLP-1 Ras: semaglutide and dulaglutide; Basal insulin: insulin degludec and insulin glargine.

In this study, there was no significant difference in the incidence of serious adverse events between tirzepatide and placebo, and basal insulin, but significant difference to GLP-1 RAs. Hypoglycemia is the major SAEs, hypoglycemia definition as blood glucose < 70 mg/dL. Across all trials, the hypoglycemia risk of tirzepatide did not differ compared with placebo and GLP-1 RAs, and was lower with tirzepatide compared with basal insulin.

After consuming tirzepatide, most of the patients experienced diarrhea, nausea, vomiting, decreased appetite, constipation, injection site reactions, and nasopharyngitis. Compared with basal insulin and placebo, more frequent gastrointestinal adverse events occurred, including diarrhea, nausea, vomiting, decreased appetite, and constipation in all tirzepatide groups. When compared with the GLP-1 RAs, the tirzepatide group showed a similar risk of nausea, diarrhea, vomiting, and constipation. While tirzepatide 5 mg and 10 mg were also associated with a higher incidence of decreased appetite. Meanwhile, there were no statistically significant differences in the incidence of injection site reactions between tirzepatide and GLP-1 RAs, and basal insulin. When compared with placebo, the incidence of injection site reactions was lower in tirzepatide (5 mg and 10 mg), but no significant difference in tirzepatide (15 mg). Besides, there was no significant difference in the incidence of nasopharyngitis was noticed between tirzepatide and placebo, GLP-1 RAs, and basal insulin.

## Discussions

Tirzepatide as the first dual GIP and GLP-1 RA drug, which shown effects on hypoglycemia, body weight and cardiovascular indicators in previous studies [31–33]. Its effect on body weight could make it useful as a weight loss drug. Thus, in this meta-analysis, a systematic review to assess the weight loss efficacy and safety of tirzepatide is conducted. Based on our findings, both doses of tirzepatide (5 mg, 10 mg and 15 mg) were more effective than other drugs in reducing body weight.

With the increase of obese people, the drugs for obesity treatment has been on the rise, and most of the drugs currently used to treat obesity started out as treatments for diabetes [34]. Based on our findings, both doses of tirzepatide were more effective in reducing bodyweight compared with other drugs. In this study, we included 10 RCT (12 reports) and compared the weight loss effects of tirzepatide with placebo, insulin, and GLP-1 RAs. The results showed that tirzepatide could reduce the weight of T2DM and obese patients. This is the same as the previous study showed [32, 35]. At the same time, the network meta-analysis results of Alkhezi et al. also showed that compared with liragrutide and semaglutide, tirzepatide had better weight reduction in non-diabetes patients, and the safety results were similar without difference [36]. Furthermore, some studies found that meaningful weight loss is achieve 5 ~ 10% [37]. In our study, tirzepatide were more effective than other drugs in reducing bodyweight of 5%, 10%, or 15%. More importantly, compared with placebo, GLP-1 RAs and insulin, tirzepatide can significantly reduce their HbA1c level of patients, and the results were same with other studies [32, 38].

For the safety, a significant difference the incidence of any adverse events between tirzepatide group and placebo/GLP-1 RAs/basal insulin. This is contrary to the results of a study by Bhagavathula et al. the results shown that no significant difference the incidence of any adverse events [32]. In addition, no significant difference in the incidence of SAEs between tirzepatide and placebo, and basal insulin, but significant difference to GLP-1 RAs. Across all trials, the risk of hypoglycemia with tirzepatide did not differ compared to placebo and GLP-1 RAs, but was lower with tirzepatide than with basal insulin. In the same results has been found in the study by Karagiannis et al. that incidence of serious adverse events did not differ between any of the tirzepatide doses and any comparator [35].

Gastrointestinal adverse events were the most common adverse events in all groups. In this study, the incidence of gastrointestinal adverse events, including diarrhea, nausea, vomiting, and decreased appetite, and the incidence of diarrhea, nausea, vomiting, and constipation were similar when comparing tirzepatide with GLP-1 RAs. However, in comparison with placebo or basal insulin, tirzepatide increased the odds of diarrhea, nausea, vomiting, decreased appetite, and constipation. The results were the same as this study [35]. The clinical trials of SURPASS reported on the gastrointestinal system, and nausea, diarrhea and vomiting were the most common AEs [17]. The results from other studies found that GLP-1 receptor agonists are often accompanied by nausea, emesis, and undesired anorexia. Importantly, the hypophagic and emetic effects of GLP-1 receptor agonists are caused by activation of central GLP-1 receptors [39]. Gastrointestinal side effects of high-dose GLP-1 RAs and co-agonists occurred in 30% ~ 70% of patients, mostly arising within the first 2 weeks of the first dose, being mild or moderate in severity, and transient [40]. A study found the incidence of gastrointestinal bleeding occurred most frequently 0 ~ 4 weeks after the first dose and was higher for the 15 mg tirzepatide group [41].

In addition, all the included studies were RCT, and some studies have a high bias risk for research because they did not have detailed randomization methods and allocation concealment or open-label. Of these, this may affect the positive outcomes. Moreover, this study could have a higher effect on weight loss due to participants of the SURMOUNT-1 study not having T2DM and the SURPASS J-Combo study with only Japanese populations. Sensitive analysis results showed that statistical heterogeneity was decreased after removing the SURMOUNT-1 study and the SURPASS J-Combo study, but the statistical effect did not change.

There are limitations in the current study. First, as all the RCTs involved the pharmaceutical industry, the positive outcomes should be interpreted cautiously [42, 43]. Second, only one study focused on the obesity patients in all 10 RCTs (12 reports), and RCTs have generally carried out in selected populations of T2DM or obesity patients. More research is suggested in the available guidance. Fortunately, 20 additional trials [17, 44, 45] aiming to investigate the efficacy of tirzepatide in the clinical setting of T2DM or obesity are ongoing. We can obtain more data to analyze after these trials are completed in the near future.

## Conclusion

To sum up, this meta-analysis indicated that tirzepatide could significantly decrease body weight in T2DM and obesity patients, and it is a potential therapeutic regimen for weight-loss, but we need to be vigilant about its gastrointestinal reaction.

## Supporting information

S1 Table The data of meta-analysis. (XLSX)

S1 Checklist PRISMA 2020 checklist. (DOCX)
