# Correction: Weight loss efficiency and safety of tirzepatide: A Systematic review

**DOI:** 10.1371/journal.pone.0346588

**Published:** 2026-04-02

**Authors:** Fei Lin, Bin Yu, Baodong Ling, Guangyao Lv, Huijun Shang, Xia Zhao, Xiaoling Jie, Jing Chen, Yan Li

The word “efficiency” is incorrect in the article title. The correct title is: Weight loss efficacy and safety of tirzepatide: A Systematic review. The correct citation is: Lin F, Yu B, Ling B, Lv G, Shang H, Zhao X, et al. (2023) Weight loss efficacy and safety of tirzepatide: A Systematic review. PLoS ONE 18(5): e0285197. https://doi.org/10.1371/journal.pone.0285197.
